# Evaluation of online text‐based information resources of gynaecological cancer symptoms

**DOI:** 10.1002/cam4.7167

**Published:** 2024-04-27

**Authors:** Tracey DiSipio, Cate Scholte, Abbey Diaz

**Affiliations:** ^1^ School of Public Health The University of Queensland Brisbane Queensland Australia

**Keywords:** cultural inclusion, gynaecological cancer, gynaecological symptoms, health literacy, indigenous health, internet, patient information

## Abstract

**Background:**

Gynaecological cancer symptoms are often vague and non‐specific. Quality health information is central to timely cancer diagnosis and treatment. The aim of this study was to identify and evaluate the quality of online text‐based patient information resources regarding gynaecological cancer symptoms.

**Methods:**

A targeted website search and Google search were conducted to identify health information resources published by the Australian government and non‐government health organisations. Resources were classified by topic (gynaecological health, gynaecological cancers, cancer, general health); assessed for reading level (Simple Measure of Gobbledygook, SMOG) and difficulty (Flesch Reading Ease, FRE); understandability and actionability (Patient Education Materials Assessment Tool, PEMAT, 0–100), whereby higher scores indicate better understandability/actionability. Seven criteria were used to assess cultural inclusivity specific for Aboriginal and Torres Strait Islander people; resources which met 3–5 items were deemed to be moderately inclusive and 6+ items as inclusive.

**Results:**

A total of 109 resources were identified and 76% provided information on symptoms in the context of gynaecological cancers. The average readability was equivalent to a grade 10 reading level on the SMOG and classified as ‘difficult to read’ on the FRE. The mean PEMAT scores were 95% (range 58–100) for understandability and 13% (range 0–80) for actionability. Five resources were evaluated as being moderately culturally inclusive. No resource met all the benchmarks.

**Conclusions:**

This study highlights the inadequate quality of online resources available on pre‐diagnosis gynaecological cancer symptom information. Resources should be revised in line with the recommended standards for readability, understandability and actionability and to meet the needs of a culturally diverse population.

## 1 INTRODUCTION

Gynaecological cancers start in the female reproductive system or genitals and include cervical, ovarian, fallopian tube, primary peritoneal, uterine/endometrial, vaginal and vulvar cancer. An estimated 6700 women will be diagnosed with gynaecological cancer in Australia in 2022 with a projected age‐standardised incidence of 42 cases per 100,000.^1^ Uterine, ovarian and cervical cancers have remained in the top 20 most commonly diagnosed cancers among Australian females for 40 years (1982 and 2022).^2^ Between 2014 and 2018, 71% of Australians diagnosed with gynaecological cancer were alive 5 years after diagnosis, with uterine cancer having the highest 5‐year survival rate (83%) and ovarian cancer having the lowest (48%). Five‐year cancer survival rates decline with more advanced disease at diagnosis. The burden of gynaecological cancers is disproportionately shared among the Australian population. National data indicate that Aboriginal and Torres Strait Islander women are more likely to be diagnosed and die from gynaecological cancers than non‐Indigenous women.^1^, ^3^, ^4^

While Australia has a national screening program for cervical cancer, timely diagnosis of other gynaecological cancers largely relies on the early identification of symptoms. The Model of Pathways to Treatment describes the events and processes that occur along the pathway to cancer diagnosis, identifying potential barriers and facilitators to diagnosis and treatment across four intervals: appraisal, help‐seeking, diagnostic and pre‐treatment.^5^ Here we are interested in the intervals prior to diagnosis. Documented barriers for appraising gynaecological cancer symptoms include experiencing non‐specific symptoms (e.g. gastrointestinal symptoms), normalisation of symptoms (e.g. attributing them to age, a side effect of hormonal contraception), self‐management of symptoms (e.g. lifestyle changes), a perception of good health (e.g. normal findings from a recent cancer screening).^6^, ^7^, ^8^ Help‐seeking may be delayed for several reasons, such as symptom embarrassment, concerns about potential judgement from providers (including wasting the doctor's time), access to care (including financially), competing time demands (e.g. work, carer duties) and unmet information needs (i.e. feeling unprepared to know what to ask or where to go for information).^6^, ^7^, ^8^, ^9^

Although less is known about the facilitators for appraisal and help‐seeking, a potentially modifiable factor is awareness that symptoms may be serious; knowledge which may be sought via available health information.^7^, ^8^ In fact, a key goal of the Australian Cancer Plan is to improve and build the cancer health literacy of the Australian population.^10^ Health literacy involves the ability of an individual to navigate the healthcare system and make health decisions; this includes the use of electronic technology.^10^ Over 80% of Australians go online first for health information and 73% have already used the internet to research a health issue before visiting their doctor, although only 6% manage to find an online health source that they trust.^11^, ^12^ The Australian population is diverse and, therefore, health information resources need to be readable, understandable and actionable by people from across different cultural and socio‐economic backgrounds. Ideally, such resources would be co‐designed with end users to ensure resources are more likely to meet their needs. End users may include a range of stakeholders including healthcare consumers, families and health professionals.^13^

After a review of the current literature, there has been no formal assessment of the availability and accessibility of information resources on gynaecological cancer symptoms for the Australian population, nor specifically the availability of resources that are culturally relevant to Aboriginal and Torres Strait Islander people, who carry the greatest burden of gynaecological cancers. Therefore, this study aims to identify and evaluate the quality of Australian online text‐based patient information resources regarding gynaecological cancer symptoms.

## 2 METHODS

### 2.1 Positionality of authors

The research team acknowledge that our individual and shared values, perspectives and experiences can shape research processes. Therefore, we recognise the value of reflexivity in research. The authorship team includes three non‐Indigenous Australian researchers, including two mid‐career and one early career public health researchers; two of whom are epidemiologists. Content areas of expertise include gynaecological cancer care, supportive care in cancer, information needs in people affected by cancer, Indigenous health, health services gaps and health inequities. The project team also included an Aboriginal research assistant.

### 2.2 Search strategy and resource selection

Two search strategies were employed to identify relevant health information resources about gynaecological cancer symptoms: (1) a targeted website search and (2) a Google search.

*Targeted website search*: A comprehensive list of Australian government and non‐government health websites that provide information, advice or services related to cancer, gynaecological health or women's health was compiled by the research team (Table 1). Multiple searches were conducted within each website using their inbuilt search function and imputing one keyword per search to identify relevant sub‐pages or embedded documents (collectively referred to as resources). Keywords included gynaecology‐, cancer‐ and symptom‐specific terms (Table 2). Here less than 50 resources were identified from a single search, all were assessed for eligibility. Otherwise, eligibility continued to be assessed beyond the first 50 resources until either all had been assessed or 20 consecutive resources were deemed ineligible. This pragmatic approach was adapted to ensure the most relevant resources were considered, while also limiting time spent on irrelevant resources.^14^ These searches were conducted between August and September 2022.

**TABLE 1:** Targeted websites.

Australian Government Websites	
Better Health Channel	https://www.betterhealth.vic.gov.au
Cancer Australia	https://www.canceraustralia.gov.au
Cancer Institute NSW	https://www.cancer.nsw.gov.au
Health Direct	https://www.healthdirect.gov.au
ACT Health	https://health.act.gov.au
SA Health	https://www.sahealth.sa.gov.au/wps/wcm/connect/public+content/sa+health+internet
VIC Health	https://www.vichealth.vic.gov.au
NSW Health	https://www.health.nsw.gov.au
NT Health	https://health.nt.gov.au/homepage
QLD Health	https://www.health.qld.gov.au
TAS Department of Health	https://www.health.tas.gov.au
WA Health	https://ww2.health.wa.gov.au

Non‐Government Websites	
Cancer Council	https://www.cancer.org.au
Cancer Council ACT	https://actcancer.org
Cancer Council NSW	https://www.cancercouncil.com.au
Cancer Council NT	https://www.cancer.org.au/nt
Cancer Council QLD	https://cancerqld.org.au/index.php
Cancer Council SA	https://www.cancersa.org.au
Cancer Council TAS	https://www.cancer.org.au/tas
Cancer Council VIC	https://www.cancervic.org.au
Cancer Council WA	https://cancerwa.asn.au
Chris O'Brien Lifehouse	https://www.mylifehouse.org.au
Cure Cancer	https://www.curecancer.com.au
Daffodil Centre	https://daffodilcentre.org
Jean Hailes	https://www.jeanhailes.org.au
VCCC Alliance	https://vcccalliance.org.au

Abbreviations: ACT, Australian Capital Territory; NSW, New South Wales; NT, Northern Territory; QLD, Queensland; SA, South Australia; TAS, Tasmania; VCCC, Victorian Comprehensive Cancer Centre; VIC, Victoria; WA, Western Australia.

**TABLE 2:** Search strategy.

Australian Government and Non‐Government Websites
Gynaecological symptoms, vaginal, abdominal, pelvic, fatigue, weight loss, weight gain, change in bowel habits, change in bladder habits, vulval lump, vaginal lump ‘gynaecological symptoms’, ‘vaginal pain’, ‘abdominal pain’, ‘pelvic pain’, ‘fatigue,’ ‘weight loss’, ‘weight gain’, ‘change in bowel habits’, ‘change in bladder habits’, ‘vulval lump’, ‘vaginal lump’

Google Search
(cervical OR uterine OR endometrial OR ovarian OR vagin* OR vulv* OR gynaecologic* OR abdominal OR pelvic) AND (symptom* OR bleeding OR discomfort OR ‘weight gain’ OR ‘weight loss’ OR pain OR bloating OR bowel OR bladder OR urin* OR lump OR mass OR incontinence OR ulceration OR pressure)

*Google search*: An advanced Google search was conducted on 21 November 2022. This search engine was selected as it is the most used in Australia.^14^ Results were limited to the English language and the region of Australia. The results displayed a title, web link and description, referred to as a ‘snippet.’ In this study, 10 snippets were listed on each results page. The first five pages of snippets were screened to identify potentially relevant resources, and screening continued until there were two consecutive pages that did not contain any potentially relevant resources. Snippets were screened to identify potentially relevant resources, which were then retrieved and reviewed against the eligibility criteria.

Eligibility criteria were systematically applied. Online text‐based resources were deemed eligible if they were published by a reliable Australian health authority, such as a government or non‐government health organisation or health service, were consumer‐focused and included any level of information about gynaecological cancer symptoms. Other information resources, such as media articles, research papers and opinion pieces (columns, blogs) were excluded, as were healthcare provider‐focused information resources. Screening was completed by one investigator (CS) and, where there was uncertainty, discussed with another investigator (TD and/or AD) to determine eligibility.

### 2.3 Evaluation of patient information resources

A data extraction form was created and piloted by two investigators (TD and CS) to ensure that key information to address the research objectives was captured. One investigator (CS) assessed eligible resources for content, readability, understandability and actionability. An Aboriginal researcher (ZG) assessed eligible resources for cultural relevance to Aboriginal and Torres Strait Islander populations. Senior investigators (TD and AD) resolved queries through discussion and checked data extraction for clarity and accuracy.

#### 2.3.1 Content summary

The primary focus of included resources was categorised as gynaecological cancers, gynaecological health, general cancer and general health symptoms. The specific pre‐diagnosis symptoms that were identified and/or discussed by the resources were summarised into broad symptom groups: abnormal or persistent vaginal bleeding, abdominal discomfort, changes in bowel and bladder habits, unexplained vulval symptoms and other general symptoms.

#### 2.3.2 Readability

Two measures of readability were used in this evaluation: (1) the Simple Measure of Gobbledygook (SMOG) estimates the years of education (i.e. school grade) required to comprehend the words used in the text based on the number of words with three syllables,^2^, ^15^, ^16^ and the Flesch Reading Ease (FRE) which calculates a score based on sentence length and number of syllables to determine the reading difficulty (score 0 = most difficult, score 100 = easiest).^15^, ^17^, ^18^ Multiple readability tools are often used to limit bias, as there is variability in the leniency of readability tools.^19^ A grade 8 reading level is recommended for health resources in Australia^20^ and an FRE score of 60 or higher (60–69 standard/average to 90–100 very easy to read) is deemed readable to the general population in Australia.^14^, ^21^ As such, these benchmarks were used in this evaluation to categorise the resources as readable or not.

An online calculator was used to conduct the readability evaluations (https://readabilityformulas.com). The online calculator requires a minimum of 150 words and a maximum of 3000 words from the text.^22^ Where possible, all text relevant to gynaecological symptoms was included. Where relevant text exceeded 3000 words, extracts from at least three different sections were included, towards the start, middle and end of the relevant text. Where the relevant word count was less than 150 words, the relevant text was copied until the minimum word count was reached.

#### 2.3.3 Understandability and actionability

The Patient Education Materials Assessment Tool (PEMAT) was used to systematically evaluate understandability and actionability of the included resources.^23^ Understandability refers to the ability of users of diverse backgrounds and health literacy levels to process and explain key messages of the resources. Actionability refers to the ability of users of diverse backgrounds and health literacy levels to identify action steps or advice for the benefit of their own health based on the information provided in the resource.

PEMAT is a validated tool that includes 19 items for understandability and seven items for actionability.^23^, ^24^, ^25^ As per the PEMAT Handbook,^23^ one investigator answered each item as yes (score = 1) or no (score = 0) and the item scores were summed and divided by the total number of items to produce a score for the understandability and actionability constructs separately. These scores were expressed as a percentage (ranging 0% = not at all understandable/actionable and 100% = easily understood/actionable). A score of 70% and over identified a resource as ‘actionable’ and ‘understandable’.^26^

#### 2.3.4 Cultural inclusivity

There is currently no validated measure of the cultural relevance of patient health information resources to Aboriginal and Torres Strait Islander people. To our knowledge, only one approach has been used previously.^27^ A team of Aboriginal and Torres Strait Islander and non‐Indigenous researchers (including investigator AD) with experience working in Indigenous health research devised seven items to assess patient information resources for cultural inclusivity. While no benchmark was provided to aid in a formal evaluation, the measure provides an indication of whether resources could be considered specifically for or inclusive of Aboriginal and Torres Strait Islander people. The seven items were

1. Does the resource include images (photos, animations, videos) of Aboriginal and Torres Strait Islander peoples?
2. Does the resource include data relevant to Aboriginal and Torres Strait Islander peoples?
3. Does the resource include Aboriginal and Torres Strait Islander designs/artwork?
4. Does the resource articulate evidence of leadership, involvement and/or governance by Aboriginal and Torres Strait Islander peoples/communities in its design/development?
5. Is the resource available in Aboriginal and Torres Strait Islander languages?
6. Is the language used appropriate, relatable and relevant for Aboriginal and Torres Strait Islander peoples?
7. Does the resource include a contact (e.g. phone number/website) for further support for Aboriginal and Torres Strait Islander peoples?

As this tool has not been validated and no benchmark determined, we somewhat arbitrarily categorised resources into three groups: not inclusive (less than 3 items were met), moderately inclusive (3–5 items were met) and inclusive (6–7 items were met).

## 3 RESULTS

**FIGURE 1:**
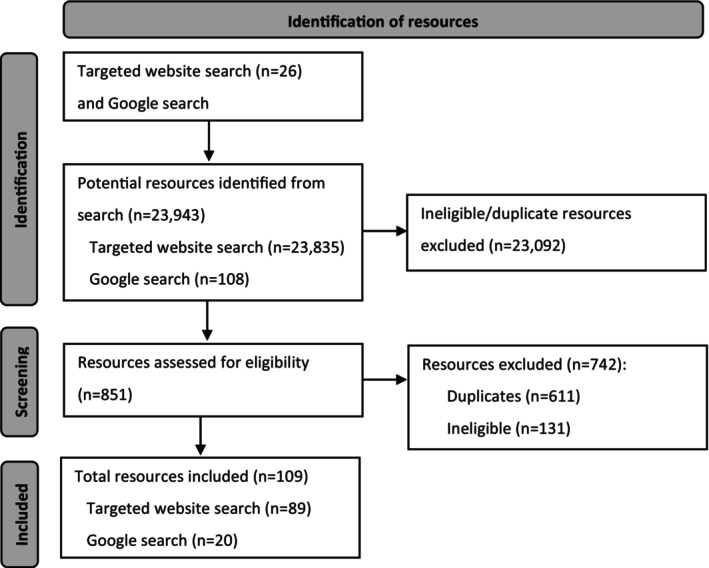
Flow diagram of resource identification and inclusion.

As illustrated in Figure 1, 23,943 potential resources were initially identified through the two search strategies. The snippets of each were assessed against the eligibility criteria and 23,092 were initially excluded. A full review of the remaining 851 resources was conducted, which resulted in a further 611 resources being excluded as duplicates and 131 excluded for not meeting eligibility criteria. The remaining 109 resources are included in this evaluation. Resources were from 25 organisations, agencies or services, including government (*n* = 8), non‐government (*n* = 8) and public or private health services (*n* = 9) (Table 3). Fourteen of the 109 resources were available in PDF printable format, and the remaining 95 were print‐friendly web pages. All were presented in colour. Weblinks to included resources are available in Data S1.

**TABLE 3:** Summary of quality evaluation for included resources.

Characteristic	Resources	
	*n*	%
Organisation type		
Government Agency/Sponsored, *n* = 8	44	40
Non‐government organisation, *n* = 8	51	47
Hospital/Service/Specialist, *n* = 9	14	13
Overall content		
Gynaecological cancers	83	76
Cancer	10	9
Gynaecological health	10	9
General health	6	6
Symptom topics^a^		
Abnormal or persistent bleeding	79	72
Abdominal discomfort	82	75
Changes in bowel or bladder habits	63	58
Unexplained vulval symptoms	52	48
Other general symptoms	64	59
SMOG readability index^b^		
Grade 6	1	1
Grade 7	5	5
Grade 8	11	10
Grade 9	17	16
Grade 10	27	25
Grade 11	23	21
Grade 12	10	9
College level and above	15	14
Flesch Reading Ease^c^		
Very easy (90–100)	0	0
Easy (80–89)	0	0
Fairly easy (70–79)	1	1
Standard (60–69)	18	17
Fairly difficult (50–59)	30	28
Difficult (30–49)	50	46
Very confusing (0–29)	10	9
PEMAT understandability		
70% and over	104	95
PEMAT actionability		
70% and over	14	13
Cultural inclusivity (0–7)		
0 items	85	78
1–2 items	19	17
3–5 items	5	5
6–7 items	0	0

	Mean	(Minimum, maximum)
Number of graphics	1	0, 20
SMOG readability index (5–18)^b^	10.4	5.6, 17.1
Flesch Reading Ease (0–100)^c^	47.9	11.0, 77.8
PEMAT Understandability (0–100)^d^	84.7	58, 100
PEMAT Actionability (0–100)^d^	48.1	0, 80
Cultural inclusivity (0–7)^e^	—	0, 5

Abbreviations: FRE, Flesch Reading Ease; PEMAT, Patient Education Materials Assessment Tool; SMOG, Simple Measure of Gobbledygook.

^a^ The counts exceed 100% as resources could address multiple symptom groups.

^b^ SMOG reading level of grade 8 and below is recommended.

^c^ FRE score of 60 or higher is considered easy to read by the general public.

^d^ PEMAT score of 70 or higher indicates the content is understandable and/or actionable.

^e^ Cultural inclusivity of 3–5 items is considered moderately inclusive and 6–7 items inclusive; due to the small number of resources meeting any cultural item, a mean was not calculated.

### 3.1 Content summary

A summary of the evaluation of included resources is available in Table 3. Three‐quarters (76%) of resources focused on gynaecological cancers, 9% on cancer symptoms, 9% on gynaecological health and 6% on general health. The majority (78%) of resources provided information on more than one symptom group. The most common symptom group identified was abdominal discomfort (75%), followed by abnormal/persistent bleeding (72%), general symptoms (59%), changes in bowel and/or bladder habits (58%) and vulval symptoms (48%).

### 3.2 Readability

The mean SMOG score was 10.4 (range 5.6–17.1), equating to a required reading level of grade 10, and the mean FRE score was 47.9 (range 11.0–77.8) denoting resources were on average ‘difficult to read’ (Tables 3 and 4). The a priori benchmarks were set at a grade 8 reading level based on the SMOG and a FRE score of 60 or higher. In this study, 16% of resources received a SMOG score equivalent to grade 8 or below, 18% of resources had a FRE score of 60 or higher (standard/average or easier) (Table 3) and 12% of resources were deemed readable using both SMOG and FRE measures (Table 4).

**TABLE 4:** Evaluation of included resources.

No.	Resource	Content summary									Readability				PEMAT^c^		Cultural inclusivity^d^						
		Format	Focus	Includes a labelled picture of anatomy	Abnormal or persistent vaginal bleeding	Abdominal discomfort	Changes in bowel or bladder habits	Unexplained vulval symptoms	Other general symptoms	Number of graphics/videos	School grade reading level (SMOG)	SMOG grade^a^	FLESCH Reading Ease (0–100)^b^	FLESCH category	Understandability score (100%)	Actionability score (100%)	Images	Relevant data	Designs or artwork	Involvement in design	Available in other languages	Appropriate language	Contact for further support
Better Health Channel (Government Agency/Sponsored)																							
1	Cervical cancer	Webpage	GC	✕	✓	✓	✕	✕	✕	0	10.6	G10	51.4	FD	85	40	✕	✕	✕	✕	✕	✕	✕
2	Cancer of the uterus	Webpage	GC	✓	✓	✓	✓	✕	✓	1	10.1	G10	50.5	FD	94	80	✕	✕	✕	✕	✕	✕	✕
3	Ovarian cancer	Webpage	GC	✕	✕	✓	✓	✕	✓	0	11.1	G11	41.8	D	83	67	✕	✕	✕	✕	✕	✕	✕
4	Vaginal cancer	Webpage	GC	✕	✓	✓	✓	✓	✕	0	12.8	CL	36.9	D	85	40	✕	✕	✕	✕	✕	✕	✕
5	Vulvar cancer	Webpage	GC	✕	✓	✕	✓	✓	✓	0	10.0	G10	48.6	D	85	40	✕	✕	✕	✕	✕	✕	✕
6	Vaginal bleeding	Webpage	GH	✕	✓	✕	✕	✕	✕	0	10.0	G10	52.6	FD	77	40	✕	✕	✕	✕	✕	✕	✕
7	Menstruation pain	Webpage	GH	✕	✓	✕	✕	✕	✕	0	14.3	CL	20.4	VC	67	40	✕	✕	✕	✕	✕	✕	✕
8	Abdominal pain in adults	Webpage	Gen	✕	✕	✓	✓	✕	✓	0	10.0	G10	59.0	FD	85	40	✕	✕	✕	✕	✕	✕	✕
9	Bowel motions	Webpage	Gen	✕	✕	✕	✓	✕	✕	0	9.1	G9	49.7	D	83	40	✕	✕	✕	✕	✕	✕	✕
10	Fatigue	Webpage	Gen	✕	✕	✕	✕	✕	✓	0	12.2	CL	40.7	D	69	40	✕	✕	✕	✕	✕	✕	✕
Cancer Australia (Government Agency/Sponsored)																							
11	Gynaecological cancer symptoms and diagnosis	Webpage	GC	✕	✓	✓	✓	✓	✓	0	11.3	G11	41.0	D	78	40	✕	✕	✕	✕	✕	✕	✕
12	Cervical cancer symptoms	Webpage	GC	✕	✓	✓	✕	✓	✓	0	11.6	G12	42.5	D	80	40	✕	✕	✕	✕	✕	✕	✕
13	Uterine cancer symptoms	Webpage	GC	✕	✓	✓	✓	✓	✕	0	9.7	G9	49.9	D	78	40	✕	✕	✕	✕	✕	✕	✕
14	Endometrial cancer symptoms	Webpage	GC	✕	✕	✓	✓	✕	✕	0	9.1	G9	46.5	D	78	40	✕	✕	✕	✕	✕	✕	✕
15	Ovarian cancer symptoms	Webpage	GC	✕	✕	✓	✓	✕	✓	0	10.1	G10	50.9	FD	78	40	✕	✕	✕	✕	✕	✕	✕
16	Ovarian cancer awareness	Webpage	GC	✕	✕	✓	✓	✕	✓	1	12.9	CL	32.1	D	92	40	✕	✕	✕	✕	✕	✕	✕
17	Managing physical changes due to ovarian cancer (and subpages)	Webpage	GC	✕	✕	✕	✓	✕	✓	0	8.5	G9	51.9	FD	78	40	✕	✕	✕	✕	✕	✕	✕
18	Vaginal cancer symptoms	Webpage	GC	✕	✓	✓	✓	✓	✕	0	8.0	G8	62.8	S	83	40	✕	✕	✕	✕	✕	✕	✕
19	Managing physical changes due to vulval cancer	Webpage	GC	✕	✕	✓	✓	✕	✓	0	8.3	G8	52.0	FD	83	40	✕	✕	✕	✕	✕	✕	✕
20	Fallopian cancer symptoms	Webpage	GC	✕	✓	✓	✓	✕	✕	0	15.9	CL	19.0	VC	78	40	✕	✕	✕	✕	✕	✕	✕
21	Cancer won't wait	Webpage	C	✕	✓	✓	✓	✕	✓	0	8.2	G8	58.4	FD	58	40	✕	✕	✕	✕	✕	✕	✕
Cancer Institute NSW (Government Agency/Sponsored)																							
22	Cervical cancer—noticing symptoms	Webpage	GC	✕	✓	✓	✕	✕	✕	0	8.6	G9	60.9	S	92	60	✕	✕	✕	✕	✕	✕	✕
23	Cervical cancer—seeing a GP	Webpage	GC	✕	✓	✕	✕	✕	✕	0	7.7	G8	67.1	S	83	60	✕	✕	✕	✕	✕	✕	✕
24	Uterine cancer—noticing symptoms	Webpage	GC	✕	✓	✓	✓	✓	✓	0	9.5	G10	53.1	FD	80	80	✕	✕	✕	✕	✕	✕	✕
25	Ovarian cancer—noticing symptoms	Webpage	GC	✕	✕	✓	✕	✕	✕	0	7.1	G7	68.8	S	80	80	✕	✕	✕	✕	✕	✕	✕
26	Vaginal cancer—noticing symptoms	Webpage	GC	✕	✓	✓	✓	✓	✕	0	8.1	G8	61.2	S	80	80	✕	✕	✕	✕	✕	✕	✕
27	Vulval cancer—noticing symptoms	Webpage	GC	✕	✓	✕	✕	✓	✕	0	6.8	G7	63.1	S	80	80	✕	✕	✕	✕	✕	✕	✕
Health Direct (Government Agency/Sponsored)																							
28	Cervical cancer	Webpage^e^	GC	✕	✓	✓	✕	✕	✓	0	10.7	G11	48.1	D	85	20	✕	✕	✕	✕	✕	✕	✕
29	Cancer of the uterus	Webpage	GC	✕	✓	✓	✓	✕	✓	0	9.2	G9	53.7	FD	85	60	✕	✕	✕	✕	✕	✕	✕
30	Endometrial cancer	Webpage^e^	GC	✕	✕	✓	✓	✓	✓	0	10.9	G11	43.6	D	92	40	✕	✕	✕	✕	✕	✕	✕
31	Ovarian cancer	Webpage	GC	✕	✓	✓	✓	✕	✓	0	10.2	G10	46.1	D	69	60	✕	✕	✕	✕	✕	✕	✕
32	Vaginal bleeding	Webpage^e^	GH	✕	✓	✕	✕	✕	✕	0	10.3	G10	54.1	FD	92	60	✕	✕	✕	✕	✕	✕	✕
33	Bleeding between periods	Webpage^e^	GH	✕	✓	✕	✕	✕	✕	0	10.4	G10	48.8	D	92	60	✕	✕	✕	✕	✕	✕	✕
34	Bleeding after menopause	Webpage^e^	GH	✕	✓	✕	✕	✕	✕	0	8.9	G9	60.3	S	85	40	✕	✕	✕	✕	✕	✕	✕
35	Painful periods	Webpage^e^	GH	✕	✓	✕	✕	✕	✕	1	10.1	G10	54.2	FD	81	60	✕	✕	✕	✕	✕	✕	✕
36	Painful sex for women	Webpage^e^	GH	✕	✕	✓	✕	✕	✕	0	9.8	G10	51.7	FD	92	80	✕	✕	✕	✕	✕	✕	✕
37	Abdominal pain	Webpage^e^	Gen	✕	✕	✓	✕	✕	✓	0	10.5	G11	49.7	D	92	60	✕	✕	✕	✕	✕	✕	✕
38	What causes abdominal pain?	Webpage^e^	Gen	✕	✕	✓	✕	✕	✕	0	9.5	G10	52.7	FD	92	40	✕	✕	✕	✕	✕	✕	✕
39	Fatigue	Webpage	Gen	✕	✕	✕	✕	✕	✓	0	8.7	G9	60.3	S	92	60	✕	✕	✕	✕	✕	✕	✕
Healthy WA (Government Agency/Sponsored)																							
40	Cervical cancer	Webpage	GC	✓	✓	✓	✕	✕	✕	1	9.8	G10	52.1	FD	100	60	✕	✕	✕	✕	✕	✕	✕
NT Health (Government Health Department)																							
41	Cervical cancer	Webpage	GC	✕	✓	✕	✕	✓	✓	0	11.6	G12	41.3	D	85	40	✕	✕	✕	✕	✕	✕	✕
42	Ovarian cancer	Webpage	GC	✕	✓	✓	✓	✕	✓	0	14.1	CL	23.7	VC	92	20	✕	✕	✕	✕	✕	✕	✕
QLD Health (Government Health Department)																							
43	Women's health: cancer (cervical and ovarian)	Webpage	GC	✕	✕	✓	✓	✓	✓	1	10.3	G10	49.7	D	69	40	✕	✕	✕	✕	✕	✕	✕
SA Health (Government Health Department)																							
44	Cancer	Webpage	C	✕	✕	✓	✓	✕	✓	0	9.1	G9	52.8	FD	83	40	✕	✕	✕	✕	✕	✕	✕
Cancer Councils of Australia (Non‐Government Organisation)																							
45	Gynaecological cancers	Webpage link to PDF #51, #56, #61, #70	GC	✕	✓	✓	✓	✓	✓	0	13.1	CL	28.3	VC	92	40	✕	✕	✓^f^	✕	✕	✕	✓^g^
46	Symptoms of cervical cancer	Webpage link to PDF #50, #51	GC	✕	✓	✓	✕	✕	✓	1	11.2	G11	47.4	D	89	40	✕	✕	✓^f^	✕	✕	✕	✕
47	Cervical cancer: what are the symptoms?	Webpage link to PDF #51	GC	✕	✓	✓	✕	✓	✕	1	12.0	G12	34.4	D	77	67	✕	✕	✕	✕	✕	✕	✕
48	Cervical cancer	Webpage link to PDF #50, #51	GC	✕	✓	✓	✕	✓	✓	0	10.7	G11	48.6	D	75	0	✕	✕	✓^f^	✕	✕	✕	✓
49	Cervical cancer overview	Webpage link to PDF #51	GC	✕	✓	✓	✕	✓	✕	0	9.7	G10	52.3	FD	92	40	✕	✕	✓^f^	✕	✕	✕	✓^g^
50	Cervical cancer (First Nations)	PDF	GC	✓	✓	✓	✕	✕	✕	1	7.5	G8	65.7	S	87	40	✕	✓	✓	✓	✕	✓	✓
51	Understanding cervical cancer	PDF p4‐15	GC	✓	✓	✓	✕	✓	✕	5	9.8	G10	52.0	FD	71	40	✕	✕	✕	✕	✕	✕	✕
52	Symptoms of cancer of the uterus	Webpage link to PDF #56	GC	✕	✓	✓	✓	✓	✓	1	10.7	G11	42.9	D	78	40	✕	✕	✓^f^	✕	✕	✕	✕
53	Uterine cancer	Webpage link to PDF #55, #56	GC	✕	✓	✓	✓	✓	✓	0	11.8	G12	38.7	D	83	0	✕	✕	✕	✕	✕	✕	✓
54	Uterine cancer overview	Webpage link to PDF #56, #57	GC	✕	✓	✓	✓	✓	✓	0	10.7	G11	44.5	D	92	0	✕	✕	✓^f^	✕	✕	✕	✓^g^
55	Cancer of the uterus (First Nations)	PDF	GC	✓	✓	✕	✕	✕	✕	1	8.7	G9	57.8	FD	94	40	✕	✕	✓	✓	✕	✓	✓
56	Understanding cancer of the uterus	PDF p4‐12	GC	✓	✓	✓	✓	✓	✓	5	11.1	G11	41.6	D	76	40	✕	✕	✕	✕	✕	✕	✕
57	Endometrial cancer (guide to best care)	PDF	GC	✕	✓	✓	✕	✕	✕	0	7.4	G7	64.9	S	85	80	✕	✕	✕	✕	✕	✓	✕
58	Ovarian cancer symptoms	Webpage link to PDF #61, #62, #92	GC	✕	✓	✓	✓	✕	✓	1	9.2	G9	56.9	FD	78	40	✕	✕	✓^f^	✕	✕	✕	✕
59	Ovarian cancer	Webpage link to PDF #61, #62	GC	✕	✓	✓	✓	✕	✓	0	10.8	G11	45.6	D	83	80	✕	✕	✓^f^	✕	✕	✕	✕
60	Ovarian cancer overview	Webpage link to PDF #61, #62, #92	GC	✕	✓	✓	✓	✕	✓	0	10.8	G11	45.2	D	92	80	✕	✕	✓^f^	✕	✕	✕	✓^g^
61	Understanding ovarian cancer	PDF p4‐15	GC	✓	✓	✓	✓	✕	✓	6	11.9	G12	42.4	D	71	40	✕	✕	✕	✕	✕	✕	✕
62	Ovarian cancer (guide to best care)	PDF	GC	✕	✕	✓	✓	✕	✓	0	7.3	G7	65.7	S	85	80	✕	✕	✕	✕	✕	✓	✕
63	Vaginal cancer symptoms	Webpage link to PDF #70	GC	✕	✓	✓	✓	✓	✓	0	11.3	G11	48.6	D	78	40	✕	✕	✓^f^	✕	✕	✕	✓^g^
64	What is vaginal cancer?	Webpage link to PDF #70	GC	✕	✓	✓	✓	✓	✕	0	11.2	G11	41.0	D	85	0	✕	✕	✕	✕	✕	✕	✕
65	Vaginal cancer	Webpage link to PDF #70	GC	✕	✓	✓	✓	✓	✕	0	10.4	G10	48.2	D	92	40	✕	✕	✕	✕	✕	✕	✕
66	Cancer of the vagina	Webpage link to PDF #70	GC	✕	✓	✓	✓	✓	✕	0	10.0	G10	49.3	D	85	40	✕	✕	✓^f^	✕	✕	✕	✓^g^
67	Vulvar cancer symptoms	Webpage link to PDF #70	GC	✕	✕	✕	✕	✓	✕	0	8.8	G9	60.2	S	78	40	✕	✕	✓^f^	✕	✕	✕	✕
68	Vulvar cancer	Webpage link to PDF #70	GC	✕	✕	✕	✕	✓	✕	0	10.0	G10	49.0	D	85	0	✕	✕	✕	✕	✕	✕	✕
69	Cancer of the vulvar	Webpage link to PDF #70	GC	✕	✕	✕	✕	✓	✕	0	9.2	G9	57.7	FD	92	40	✕	✕	✓^f^	✕	✕	✕	✓^g^
70	Understanding vulvar and vaginal Cancers	PDF p13, 37	GC	✓	✕	✕	✕	✓	✓	5	10.1	G10	51.0	FD	76	40	✕	✕	✕	✕	✕	✕	✕
71	Early detection and screening	Webpage	GC	✕	✓	✕	✓	✓	✓	0	7.8	G8	63.2	S	89	40	✕	✕	✕	✕	✕	✕	✕
72	Cancer—what to expect booklet (First Nations)	PDF p2	C	✕	✕	✕	✕	✕	✓	18	7.7	G8	68.3	S	81	60	✓	✓	✓	✕	✕	✓	✓
73	Common cancer symptoms	Webpage	C	✕	✓	✓	✓	✕	✓	0	9.0	G9	62.0	S	85	60	✕	✕	✕	✕	✕	✕	✕
74	Women and cancer	PDF p12‐14	C	✕	✓	✓	✓	✓	✓	3	9.4	G9	53.3	FD	83	40	✕	✕	✕	✕	✕	✕	✕
75	Get checked—women	Webpage	C	✕	✓	✓	✓	✓	✓	1	9.2	G9	55.1	FD	83	40	✕	✕	✓^f^	✕	✕	✕	✕
76	Understanding your body	Webpage	C	✕	✓	✓	✓	✓	✓	0	10.7	G11	47.5	D	89	40	✕	✕	✕	✕	✕	✕	✕
77	What is cancer—information for people affected by cancer	PDF	C	✕	✓	✓	✓	✓	✓	2	7.4	G7	66.0	S	87	60	✕	✕	✕	✕	✕	✕	✕
78	What is cancer—easy read	PDF	C	✕	✕	✕	✕	✕	✓	>20	5.6	G6	77.8	FE	93	80	✕	✕	✓	✕	✕	✓	✓
79	What is cancer	Webpage link to PDF #77, #78	C	✕	✓	✓	✓	✓	✓	1	7.7	G8	63.4	S	88	40	✕	✕	✓^f^	✕	✕	✕	✓^g^
Cure Cancer (Non‐Government Organisation)																							
80	Gynaecological cancer	Webpage	GC	✕	✓	✓	✓	✓	✓	1	13.6	CL	26.5	VC	85	40	✕	✕	✕	✕	✕	✕	✕
Jean Hailes (Non‐Government Organisation)																							
81	Cervical cancer	Webpage	GC	✕	✓	✓	✕	✓	✓	1	10.8	G11	46.4	D	92	40	✕	✕	✕	✕	✕	✕	✕
82	Endometrial cancer	Webpage link to PDF #56	GC	✕	✓	✓	✓	✓	✓	0	12.9	CL	26.5	VC	93	50	✕	✕	✕	✕	✕	✕	✕
83	Ovarian cancer	Webpage link to PDF #61	GC	✕	✓	✓	✓	✕	✓	1	11.4	G12	37.2	D	93	67	✕	✕	✕	✕	✕	✕	✕
84	Vaginal cancer	Webpage link to PDF #70, #89	GC	✓	✓	✓	✓	✓	✕	1	12.1	G12	37.9	D	94	40	✕	✕	✕	✕	✕	✕	✕
85	Vulval cancer	Webpage link to PDF #70	GC	✓	✓	✕	✕	✓	✕	1	10.8	G11	43.4	D	94	40	✕	✕	✕	✕	✕	✕	✕
86	Fallopian tube cancer	Webpage	GC	✕	✓	✓	✓	✓	✓	1	11.6	G12	36.8	D	94	40	✕	✕	✕	✕	✕	✕	✕
87	Painful sex	Webpage	GH	✕	✕	✓	✕	✕	✕	1	17.0	CL	11.0	VC	83	40	✕	✕	✕	✕	✕	✕	✕
88	Vulval and vaginal health	PDF	GH	✕	✕	✕	✕	✓	✕	4	8.3	G8	59.9	S	94	80	✕	✕	✕	✕	✕	✕	✕
89	The vulva	PDF	GC	✓	✕	✕	✕	✓	✕	12	10.7	G11	42.4	D	88	80	✕	✕	✕	✕	✕	✕	✕
90	Vulval and vaginal irritation	Webpage	GH	✕	✕	✓	✕	✓	✕	1	10.7	G11	45.2	D	81	60	✕	✕	✓^f^	✕	✕	✕	✕
Ovarian Cancer Australia (Non‐Government Organisation)																							
91	Signs and symptoms (webpage) with tracker (PDF) (ovarian)	Webpage with embedded PDF	GC	✕	✓	✓	✓	✕	✓	1	10.2	G10	45.1	D	83	60	✕	✕	✕	✕	✕	✕	✕
Ovarian Cancer Research Fund (Non‐Government Organisation)																							
92	Signs and symptoms (ovarian)	Webpage	GC	✕	✓	✓	✓	✓	✓	2	12.0	G12	34.4	D	87	40	✕	✕	✕	✕	✕	✕	✕
My Dr (Non‐Government Organisation)																							
93	Cervical cancer symptoms and diagnosis	Webpage	GC	✕	✓	✓	✕	✓	✕	1	9.7	G10	52.9	FD	83	60	✕	✕	✕	✕	✕	✕	✕
My Health First (Non‐Government Organisation)																							
94	A closer look at gynaecological cancers	Webpage	GC	✕	✓	✓	✓	✓	✓	1	13.2	CL	31.2	D	81	40	✕	✕	✕	✕	✕	✕	✕
Women Can (Non‐Government Organisation)																							
95	Ovarian cancer	Webpage	GC	✕	✓	✓	✓	✕	✓	0	14.0	CL	22.2	VC	92	40	✕	✕	✕	✕	✕	✕	✕
Chris O'Brien Lifehouse (Hospital/Service/Specialist)																							
96	Cervical cancer	Webpage link to PDF #51	GC	✕	✓	✓	✕	✓	✓	0	11.1	G11	46.5	D	92	60	✕	✕	✕	✕	✕	✕	✕
97	Uterine cancer	Webpage link to PDF #56	GC	✕	✓	✓	✓	✓	✕	0	11.3	G11	39.2	D	83	40	✕	✕	✕	✕	✕	✕	✕
98	Ovarian cancer	Webpage link to PDF #61	GC	✕	✓	✓	✓	✕	✓	0	9.7	G10	46.5	D	92	40	✕	✕	✕	✕	✕	✕	✕
99	Vaginal cancer	Webpage link to PDF #70	GC	✓	✓	✓	✓	✓	✓	1	9.7	G10	51.0	FD	94	60	✕	✕	✕	✕	✕	✕	✕
100	Vulval cancer	Webpage link to PDF #70	GC	✓	✕	✕	✕	✓	✕	1	8.4	G8	56.6	FD	100	40	✕	✕	✕	✕	✕	✕	✕
The Royal Women's Hospital (Hospital/Service/Specialist)																							
101	Cervical cancer	Webpage	GC	✕	✓	✓	✕	✕	✓	0	9.9	G10	54.5	FD	92	60	✕	✕	✕	✕	✕	✕	✕
102	Ovarian cancer	Webpage	GC	✕	✕	✓	✓	✕	✓	0	10.5	G11	50.5	FD	92	80	✕	✕	✕	✕	✕	✕	✕
Mater (Hospital/Service/Specialist)																							
103	Ovarian cancer	Webpage	GC	✕	✓	✓	✓	✕	✓	5	13.9	CL	25.5	VC	77	60	✕	✕	✕	✕	✕	✕	✕
Hunter Valley Oncology (Hospital/Service/Specialist)																							
104	Ovarian Cancer Treatment Newcastle	Webpage	GC	✕	✓	✓	✓	✕	✓	3	11.6	G12	40.7	D	73	60	✕	✕	✕	✕	✕	✕	✕
Icon Cancer Centre (Hospital/Service/Specialist)																							
105	Cervical cancer	Webpage link to PDF #51	GC	✓	✓	✓	✕	✓	✕	9	17.1	CL	19.0	VC	80	40	✕	✕	✕	✕	✕	✕	✕
Cherbourg Regional Aboriginal and Islander Community Controlled Health Service (Hospital/Service/Specialist)																							
106	Ovarian cancer: symptoms, diagnosis and treatment	Webpage	GC	✕	✓	✓	✓	✕	✓	1	9.6	G10	54.8	FD	75	60	✕	✕	✓^f^	✓	✕	✓	✓
Globe Medical (Hospital/Service/Specialist)																							
107	Ovarian cancer	Webpage	GC	✕	✓	✓	✓	✕	✓	1	13.9	CL	32.0	D	93	50	✕	✕	✕	✕	✕	✕	✕
Gynae‐oncologist (Hospital/Service/Specialist)																							
108	Cervical cancer	Webpage	GC	✕	✓	✓	✓	✕	✕	1	10.9	G11	44.6	D	85	60	✕	✕	✕	✕	✕	✕	✕
109	Cervical cancer	Webpage	GC	✕	✓	✕	✕	✕	✕	0	9.4	G9	49.2	D	85	40	✕	✕	✕	✕	✕	✕	✕

*Note*: Content summary: C, Cancer; GC, Gynaecological cancers; Gen, General health; GH, Gynaecological health. Readability: CL, College level; G, Grade; D, Difficult; VD, Very difficult; FD, Fairly difficult; S, Standard; VC, Very confusing.

Abbreviations: FRE, Flesch Reading Ease; GP, General Practitioner; No., Number; PEMAT, Patient Education Materials Assessment Tool; SMOG, Simple Measure of Gobbledygook.

^a^ SMOG reading level of grade 8 and below is recommended.

^b^ FRE score of 60 or higher is considered easy to read by the general public.

^c^ PEMAT score of 70 or higher indicates the content is understandable and/or actionable.

^d^ Cultural inclusivity of 3–5 items is considered moderately inclusive and 6–7 items as inclusive.

^e^ Webpage also linked to tools (e.g. symptom checker, service finder, question builder) which were not included in the formal health literacy assessment although these are likely to increase the actionability of the resource.

^f^ Small image included, in header/footer and typically alongside an Acknowledgement of Country.

^g^ Link to further information/support provided in the website menu rather than on the webpage itself.

### 3.3 Understandability

The mean PEMAT understandability score was 84.7% (range 58%–100%) (Table 2). Almost all resources (95%) met or exceeded the 70% benchmark used for this evaluation (Table 4). Of note, only 39% of resources used visual aids, including 14 resources which included labelled pictures of anatomy.

### 3.4 Actionability

The mean PEMAT actionability score was 48.1% (range 0%–80%) (Table 3). Only 13% of resources received an actionability score of 70% or higher, thus meeting the benchmark set for this evaluation (Table 4). The most common actionable advice provided to the readers was to consult their general practitioner (GP) if symptoms arose and/or persisted.

### 3.5 Cultural inclusivity

Five resources were considered moderately culturally inclusive for Aboriginal and Torres Strait Islander people (Table 4). Four of these resources were explicitly Aboriginal and Torres Strait Islander‐focused and related to cervical cancer, uterine cancer, cancer (Cancer Councils of Australia, resources #50, #55, #72) and ovarian cancer [Cherbourg Regional Aboriginal and Islander Community Controlled Health Services (CRAICCHS), resource #106]. The other resource was an easy‐read booklet (Cancer Councils of Australia, resource #78). A further 19 resources met one or two items each for cultural inclusivity. No resource met six or more items to be considered culturally inclusive.

### 3.6 Overall evaluation and model resources

Overall, no individual resource met all the benchmarks for readability, understandability, actionability and cultural inclusivity of Aboriginal and Torres Strait Islander peoples. Six resources met the benchmarks for readability, understandability and actionability and identified a range of gynaecological symptoms (covering between one and four of the five symptom groups). These resources were published by the Cancer Institute NSW (resources #25, #26, #27) and the Cancer Councils of Australia (resources #57, #62, #78). One of these six resources was deemed to be moderately culturally inclusive (resource #78).

## 4 DISCUSSION

This study identified and evaluated the quality of Australian, online text‐based, information resources on gynaecological cancer symptoms. Given that 80% of Australians access health information online,^11^ it is important for women to be able to source online information that is readable, understandable, actionable and culturally inclusive. While there was no shortage in the *quantity* of resources (*n* = 109), the *quality* varied considerably with just six resources meeting three (i.e. readable, understandable, actionable) of the four (plus cultural inclusivity) predetermined benchmarks.

Although a significant number of online information resources for gynaecological cancer symptoms are published by Australian health authorities, not all symptom groups were addressed equally. Unexplained vulval symptoms were the least described (<50% of resources mentioned vulval symptoms such as a lump, ulceration, bleeding, skin colour changes, persistent itching, burning or soreness). Women diagnosed with gynaecological cancer, and vulval cancer in particular, have identified information barriers including not knowing where to go for information, not knowing what to ask, and a preference for information from sources other than the internet, highlighting the importance of ensuring quality health resources in different formats to educate and support the diagnostic process.^7^, ^8^, ^28^

Overall, the resources identified were rated as ‘difficult’ to read by the general public with the majority not meeting the Australian recommended guidelines for readability of health resources. Our finding concurs with previous reports that the average readability of information on cancer websites globally and health websites in general are difficult to read.^29^, ^30^, ^31^ In fact, Australian heath websites have been found to be written at two to four grades higher than recommended; indeed, information on bowel and breast cancer is significantly harder to read than other conditions such as anxiety, diabetes and obesity.^32^

There was a high standard of understandability among the evaluated resources with the majority meeting this benchmark.^26^ However, there is room for further improvement as few resources use visual aids. Integrating visual aids supports content to be more easily understood, clearly directs readers to important information and increases the aesthetic appearance of information.^23^ Furthermore, the use of visual aids in health information can specifically benefit patients with limited health literacy.^23^ Conversely, there was a lack of actionable information provided with only 13% of resources meeting the benchmark.^26^ The most common action recommended was for readers to consult their GP if they are experiencing any symptoms.

The findings illustrate limited culturally inclusive resources, with only four resources written for Aboriginal and Torres Strait Islander populations. Australian research has highlighted the importance of individualising cancer information by taking into consideration demographic factors and attitudes^33^; although this finding is specific to rural cancer survivors, the conclusions are also relevant to enhancing the inclusion of Aboriginal and Torres Strait Islander people in healthcare. Aboriginal and Torres Strait Islander women experience higher incidence and mortality rates for gynaecological cancers than non‐Indigenous women, particularly for cervical cancer, although no national data are available on vaginal and vulvar cancers.^34^ Culturally inclusive information has the potential to support women during appraisal and help‐seeking intervals and subsequently improve the timeliness of cancer diagnosis and treatment.^35^ The provision of culturally relevant resources on gynaecological cancer symptoms for Aboriginal and Torres Strait Islander people may help reduce the longstanding inequities in gynaecological cancer outcomes.

### 4.1 Strengths and limitations

While this evaluation comprehensively included relevant government and non‐government‐authorised Australian websites, an international evaluation would have provided a more accurate representation of resources available to the general public. The evaluation of relevant resources was conducted with validated tools (SMOG, FRE, PEMAT) with consistent use of one online calculator, as recent evidence suggests that automated readability scores vary across calculators.^36^ These validated tools have previously been used to assess the quality of information created for Australian women following the renewal of the Cervical Screening Program.^37^ Furthermore, the FRE has been used to assess the readability of online information for women from culturally and linguistically diverse backgrounds with low health literacy.^38^ While the cultural inclusivity tool used is not validated, no alternative evaluation tools are available for use within Australia. Future research is necessary to validate this tool and to identify and evaluate other resource formats such as videos and podcasts.

### 4.2 What do the results mean?

As the identified resources were not deemed to be readable, understandable, actionable or culturally inclusive, it is important to highlight the impact this has on gynaecological cancer pathways. The Model of Pathways to Treatment illustrates the importance of patient cancer awareness and health literacy for timely help‐seeking and diagnosis.^5^ Low health literacy is associated with poorer health and higher mortality, and complex health materials act as a barrier to health.^39^ Implications for research and practice include building the cancer health literacy of the Australian population in an equitable and inclusive way, as per the Australian Cancer Care Plan.^10^ Consistent with the National Women's Health Strategy priorities, this may include, but is not limited to, educating people where to go for trustworthy information and improving awareness of gynaecological cancer symptoms.^40^ There is some evidence that interventions to promote awareness of cancer symptoms delivered to individuals and via public education campaigns may be successful,^41^ although further research is required to test the effectiveness of promoting help‐seeking behaviours,^42^ including for gynaecological cancer symptoms.^43^

Improving online health resources in accordance with readability, understandability and actionability recommendations for a diverse Australian population is required. For example, integrating visual aids improves understandability and benefits those with limited health literacy.^23^ A few identified resources included a question prompt list, which has been found to be effective during oncology treatment consultations.^44^ A question list may also be useful to ease the barrier to seeking help when women feel unprepared to know what to ask pre‐diagnosis^7^ and, as an actionable item, supports women to take steps that can benefit their own health. Ideally, resources should be (re)designed with input from end users; in doing so, resources may be more appropriate, acceptable and feasible to implement for the intended population. As there already exists many information resources about gynaecological cancer symptoms for Australian women, the first step of re‐design through a co‐design process is to identify high‐quality resources (i.e. identified here) and then work with end users to identify elements of these that need to be changed, improved or maintained.^13^ This audit of resources will form the basis of such work.

## 5 CONCLUSION

This study provides a summary of online gynaecological cancer symptom resources in Australia and highlights the inadequate quality of pre‐diagnosis resources available. Online information should be designed with particular consideration to people with low health literacy and for a culturally diverse population. A simple increase in the *quantity* of information will not solve this barrier with the internet already oversaturated; rather, the modification of existing resources to align with Australian standards for quality information is urgently needed. Finally, we advocate for health information resources to be (re)designed in collaboration with end users to ensure their suitability, both in terms of health literacy and cultural relevance.

## AUTHOR CONTRIBUTIONS

**Tracey DiSipio:** Conceptualization (equal); investigation (equal); methodology (equal); project administration (equal); supervision (equal); validation (equal); visualization (equal); writing – original draft (equal); writing – review and editing (equal). **Cate Scholte:** Data curation (equal); formal analysis (equal); investigation (equal); visualization (equal); writing – original draft (equal); writing – review and editing (equal). **Abbey Diaz:** Conceptualization (equal); investigation (equal); methodology (equal); project administration (equal); supervision (equal); validation (equal); visualization (equal); writing – review and editing (equal).

## FUNDING INFORMATION

Not applicable.

## CONFLICT OF INTEREST STATEMENT

None to declare.

## Supporting information


Data S1

## Data Availability

The data underlying this article are available in the article and in its supplementary material.
