# Supplementary material for: Evaluation of online text‐based information resources of gynaecological cancer symptoms
Source: Cancer Med. 2024 Apr 27;13(9):e7167. doi: 10.1002/cam4.7167 (PMC11053368; doi:10.1002/cam4.7167)
Supplement: Supplementary file 1 — Data S1 [file CAM4-13-e7167-s001.docx]

| Supplementary Material S1. Weblink for included resources | | |
| --- | --- | --- |
| No. | **RESOURCE** | **WEBLINK** |
| Better Health Channel (Government Agency/Sponsored) | | |
| 1 | Cervical cancer | https://www.betterhealth.vic.gov.au/health/conditionsandtreatments/cervical-cancer |
| 2 | Cancer of the uterus | https://www.betterhealth.vic.gov.au/health/conditionsandtreatments/uterine-cancer |
| 3 | Ovarian cancer | https://www.betterhealth.vic.gov.au/health/conditionsandtreatments/ovarian-cancer |
| 4 | Vaginal cancer | <https://www.betterhealth.vic.gov.au/health/conditionsandtreatments/vaginal-cancer> |
| 5 | Vulvar cancer | https://www.betterhealth.vic.gov.au/health/conditionsandtreatments/vulvar-cancer |
| 6 | Vaginal bleeding | https://www.betterhealth.vic.gov.au/health/conditionsandtreatments/vaginal-bleeding-irregular |
| 7 | Menstruation pain | <https://www.betterhealth.vic.gov.au/health/conditionsandtreatments/menstruation-pain-dysmenorrhoea> |
| 8 | Abdominal pain in adults | https://www.betterhealth.vic.gov.au/health/conditionsandtreatments/abdominal-pain-in-adults |
| 9 | Bowel motions | https://www.betterhealth.vic.gov.au/health/conditionsandtreatments/bowel-motions |
| 10 | Fatigue | <https://www.betterhealth.vic.gov.au/health/conditionsandtreatments/fatigue> |
| Cancer Australia (Government Agency/Sponsored) | | |
| 11 | Gynaecological cancers symptoms and diagnosis | https://www.canceraustralia.gov.au/cancer-types/gynaecological-cancers/symptoms-and-diagnosis |
| 12 | Cervical cancer symptoms | https://www.canceraustralia.gov.au/cancer-types/cervical-cancer/symptoms-and-diagnosis |
| 13 | Uterine cancer symptoms | https://www.canceraustralia.gov.au/cancer-types/uterine-cancer/symptoms-and-diagnosis |
| 14 | Endometrial cancer symptoms | <https://www.canceraustralia.gov.au/cancer-types/endometrial-cancer/symptoms-and-diagnosis> |
| 15 | Ovarian cancer symptoms | https://www.canceraustralia.gov.au/cancer-types/ovarian-cancer/symptoms-and-diagnosis |
| 16 | Ovarian cancer awareness | https://www.canceraustralia.gov.au/affected-cancer/cancer-types/ovarian-cancer/ovarian-cancer-awareness |
| 17 | Managing physical changes due to ovarian cancer (and subpages) | https://www.canceraustralia.gov.au/impacted-by-cancer/physical-changes |
| 18 | Vaginal cancer symptoms | https://www.canceraustralia.gov.au/cancer-types/vaginal-cancer/symptoms-and-diagnosis |
| 19 | Managing physical changes due to vulval cancer | https://www.canceraustralia.gov.au/cancer-types/vulval-cancer/impacted-by-vulval-cancer/physical-changes |
| 20 | Fallopian cancer symptoms | https://www.canceraustralia.gov.au/cancer-types/fallopian-cancer/symptoms-and-diagnosis |
| 21 | Cancer won’t wait | <https://www.canceraustralia.gov.au/cancer-wont-wait> |
| Cancer Institute NSW (Government Agency/Sponsored) | | |
| 22 | Cervical cancer - noticing symptoms | https://www.cancer.nsw.gov.au/cervical-cancer/diagnosis-and-treatment/diagnosis/noticing-symptoms |
| 23 | Cervical cancer - seeing a GP | <https://www.cancer.nsw.gov.au/about-cancer/types-of-cancer/cervical-cancer/diagnosis/seeing-a-gp> |
| 24 | Uterine cancer - noticing symptoms | https://www.cancer.nsw.gov.au/uterine-cancer/diagnosis-and-treatment/diagnosis/noticing-symptoms#1f6963c9-d490-4176-8bcd-548105b0468c |
| 25 | Ovarian cancer - noticing symptoms | https://www.cancer.nsw.gov.au/ovarian-cancer/diagnosis-and-treatment/diagnosis/noticing-symptoms |
| 26 | Vaginal cancer - noticing symptoms | https://www.cancer.nsw.gov.au/vaginal-cancer/diagnosis-and-treatment/diagnosis/noticing-symptoms |
| 27 | Vulval cancer - noticing symptoms | https://www.cancer.nsw.gov.au/vulvar-cancer/diagnosis-and-treatment/diagnosis/noticing-symptoms |
| Health Direct (Government Agency/Sponsored) | | |
| 28 | Cervical cancer | https://www.healthdirect.gov.au/cervical-cancer |
| 29 | Cancer of the uterus | <https://www.healthdirect.gov.au/cancer-of-the-uterus> |
| 30 | Endometrial cancer | <https://www.healthdirect.gov.au/endometrial-cancer> |
| 31 | Ovarian cancer | <https://www.healthdirect.gov.au/cervical-cancer> |
| 32 | Vaginal bleeding | <https://www.healthdirect.gov.au/vaginal-bleeding> |
| 33 | Bleeding between periods | <https://www.healthdirect.gov.au/bleeding-between-periods> |
| 34 | Bleeding after menopause | https://www.healthdirect.gov.au/bleeding-after-menopause |
| 35 | Painful periods | <https://www.healthdirect.gov.au/painful-periods> |
| 36 | Painful sex for women | https://www.healthdirect.gov.au/painful-sex-for-women |
| 37 | Abdominal pain | <https://www.healthdirect.gov.au/abdominal-pain> |
| 38 | What causes abdominal pain? | <https://www.healthdirect.gov.au/what-causes-abdominal-pain> |
| 39 | Fatigue | <https://www.healthdirect.gov.au/fatigue> |
| Healthy WA (Government Agency/Sponsored) | | |
| 40 | Cervical cancer | <https://www.healthywa.wa.gov.au/Articles/A_E/Cervical-cancer> |
| NT Health (Government Health Department) | | |
| 41 | Cervical cancer | https://nt.gov.au/wellbeing/cancer-services/cervical-cancer |
| 42 | Ovarian cancer | https://nt.gov.au/wellbeing/cancer-services/ovarian-cancer |
| QLD Health (Government Health Department) | | |
| 43 | Women's health: cancer (cervical and ovarian) | <https://www.qld.gov.au/health/staying-healthy/men-women/women/cancer> |
| SA Health (Government Health Department) | | |
| 44 | Cancer | https://www.sahealth.sa.gov.au/wps/wcm/connect/public+content/sa+health+internet/conditions/cancer/cancer |
| Cancer Councils of Australia (Non-Government Organisation) | | |
| 45 | Gynaecological cancers | https://cancerqld.org.au/cancer-information/types-of-cancer/gynaecological-cancers/ |
| 46 | Symptoms of cervical cancer | <https://www.cancercouncil.com.au/cervical-cancer/symptoms/> |
| 47 | Cervical cancer: what are the symptoms? | <https://www.cancersa.org.au/cancer-a-z/cervical-cancer/what-are-the-symptoms/> |
| 48 | Cervical cancer | <https://www.cancer.org.au/cancer-information/types-of-cancer/cervical-cancer> |
| 49 | Cervical cancer overview | https://www.cancervic.org.au/cancer-information/types-of-cancer/cervical_cancer/cervical-cancer-overview.html |
| 50 | Cervical cancer (First Nations) | <https://www.cancercouncil.com.au/wp-content/uploads/2023/02/Cervical-cancer-Aboriginal.pdf> |
| 51 | Understanding cervical cancer | https://www.cancer.org.au/assets/pdf/understanding-cervical-cancer-booklet |
| 52 | Symptoms of cancer of the uterus | <https://www.cancercouncil.com.au/uterine-cancer/symptoms/> |
| 53 | Uterine cancer | <https://www.cancer.org.au/cancer-information/types-of-cancer/uterine-cancer> |
| 54 | Uterine cancer overview | <https://www.cancervic.org.au/cancer-information/types-of-cancer/uterine_cancer/uterine-cancer-overview.html> |
| 55 | Cancer of the uterus (First Nations) | <https://www.cancer.org.au/assets/pdf/aboriginal-and-torres-strait-islander-cancer-of-the-uterus-factsheet> |
| 56 | Understanding cancer of the uterus | https://www.cancer.org.au/assets/pdf/understanding-uterus-cancer-booklet |
| 57 | Endometrial cancer (guide to best care) | https://www.cancer.org.au/assets/pdf/endometrial-cancer-english |
| 58 | Ovarian cancer symptoms | <https://www.cancercouncil.com.au/ovarian-cancer/symptoms/> |
| 59 | Ovarian cancer | <https://cancerqld.org.au/cancer-information/types-of-cancer/ovarian-cancer/> |
| 60 | Ovarian cancer overview | https://www.cancervic.org.au/cancer-information/types-of-cancer/ovarian_cancer/ovarian-cancer-overview.html |
| 61 | Understanding ovarian cancer | https://www.cancer.org.au/assets/pdf/understanding-ovarian-cancer-booklet |
| 62 | Ovarian cancer (guide to best care) | <https://www.cancer.org.au/assets/pdf/ovarian-cancer-english> |
| 63 | Vaginal cancer symptoms | https://www.cancercouncil.com.au/vaginal-cancer/symptoms/ |
| 64 | What is vaginal cancer? | <https://www.cancer.org.au/cancer-information/types-of-cancer/vaginal-cancer> |
| 65 | Vaginal cancer | <https://cancerqld.org.au/cancer-information/types-of-cancer/vaginal-cancer/> |
| 66 | Cancer of the vagina | https://www.cancervic.org.au/cancer-information/types-of-cancer/vulvar-vaginal-cancers/vaginal_cancer-tmp.html |
| 67 | Vulvar cancer symptoms | <https://www.cancercouncil.com.au/vulvar-cancer/symptoms/> |
| 68 | Vulvar cancer | <https://www.cancer.org.au/cancer-information/types-of-cancer/vulvar-cancer> |
| 69 | Cancer of the vulvar | https://www.cancervic.org.au/cancer-information/types-of-cancer/vulvar-vaginal-cancers/vulvar_cancer.html |
| 70 | Understanding vulvar and vaginal cancers | <https://www.cancer.org.au/assets/pdf/understanding-vulvar-and-vaginal-cancers-booklet> |
| 71 | Early detection and screening | https://www.actcancer.org/prevention/early-detection-and-screening/ |
| 72 | Cancer - what to expect booklet (First Nations) | <https://www.cancer.org.au/assets/pdf/cancer-what-to-expect#_ga=2.242348866.1711426497.1688013945-2112092584.1659074836> |
| 73 | Common cancer symptoms | <https://www.cancer.org.au/cancer-information/what-is-cancer/cancer-symptoms> |
| 74 | Women and cancer | https://www.cancercouncil.com.au/wp-content/uploads/2021/07/Women-and-Cancer.pdf |
| 75 | Get checked - women | https://www.cancer.org.au/cancer-information/causes-and-prevention/early-detection-and-screening/get-checked-women |
| 76 | Understanding your body | https://www.cancersa.org.au/prevention/finding-cancer-early/understanding-your-body/ |
| 77 | What is cancer - Information for people affected by cancer | <https://www.cancervic.org.au/downloads/CISS_factsheets/what_is_cancer.pdf> |
| 78 | What is cancer - easy read | <https://www.cancervic.org.au/downloads/CISS_factsheets/what_is_cancer_easy_read.pdf> |
| 79 | What is cancer? | <https://www.cancervic.org.au/cancer-information/what-is-cancer> |
| Cure Cancer (Non-Government Organisation) | | |
| 80 | Gynaecological cancer | https://www.curecancer.com.au/cancer-types/gynaecological-cancer |
| Jean Hailes (Non-Government Organisation) | | |
| 81 | Cervical cancer | https://www.jeanhailes.org.au/health-a-z/vulva-vagina-ovaries-uterus/gynaecological-cancers/cervical-cancer |
| 82 | Endometrial cancer | https://www.jeanhailes.org.au/health-a-z/vulva-vagina-ovaries-uterus/gynaecological-cancers/endometrial-uterine-cancer |
| 83 | Ovarian cancer | https://www.jeanhailes.org.au/health-a-z/vulva-vagina-ovaries-uterus/gynaecological-cancers/ovarian-cancer |
| 84 | Vaginal cancer | <https://www.jeanhailes.org.au/health-a-z/vulva-vagina-ovaries-uterus/gynaecological-cancers/vaginal-cancer> |
| 85 | Vulval cancer | <https://www.jeanhailes.org.au/health-a-z/vulva-vagina-ovaries-uterus/gynaecological-cancers/vulvar-cancer> |
| 86 | Fallopian tube cancer | https://www.jeanhailes.org.au/health-a-z/vulva-vagina-ovaries-uterus/gynaecological-cancers/fallopian-tube-cancer |
| 87 | Painful sex | <https://www.jeanhailes.org.au/health-a-z/sex-sexual-health/painful-sex-dyspareunia> |
| 88 | Vulval and vaginal health | https://www.jeanhailes.org.au/uploads/Fact_sheets/jh_factsheet_vulva_vaginal_health_plain_english.pdf |
| 89 | The vulva | https://assets.jeanhailes.org.au/Booklets/The_vulva.pdf |
| 90 | Vulval and vaginal irritation | <https://www.jeanhailes.org.au/health-a-z/vulva-vagina/vulval-irritation> |
| Ovarian Cancer Australia (Non-Government Organisation) | | |
| 91 | Signs and symptoms (webpage) with tracker (PDF) (ovarian) | <https://www.ovariancancer.net.au/page/134/signs-and-symptoms> |
| Ovarian Cancer Research Fund (Non-Government Organisation) | | |
| 92 | Signs and symptoms (ovarian) | https://www.ocrf.com.au/ovarian-cancer/symptoms-and-signs |
| My Dr (Non-Government Organisation) | | |
| 93 | Cervical cancer symptoms and diagnosis | <https://mydr.com.au/symptoms/cervical-cancer-symptoms-and-diagnosis/> |
| My Health 1st (Non-Government Organisation) | | |
| 94 | A closer look at gynaecological cancers | https://www.myhealth1st.com.au/health-hub/articles/gynaecological-cancers/ |
| Women Can (Non-Government Organisation) | | |
| 95 | Ovarian cancer | <https://www.womencan.org.au/gynae-cancers/ovarian-cancer> |
| Chris O’Brien Lifehouse (Hospital/Service/Specialist) | | |
| 96 | Cervical cancer | https://www.mylifehouse.org.au/for-patients/cancer-types/cervical/ |
| 97 | Uterine cancer | https://www.mylifehouse.org.au/for-patients/cancer-types/uterus/ |
| 98 | Ovarian cancer | https://www.mylifehouse.org.au/for-patients/cancer-types/ovarian/ |
| 99 | Vaginal cancer | https://www.mylifehouse.org.au/for-patients/cancer-types/vaginal/ |
| 100 | Vulval cancer | https://www.mylifehouse.org.au/for-patients/cancer-types/vulva/ |
| The Royal Women’s Hospital (Hospital/Service/Specialist) | | |
| 101 | Cervical cancer | https://www.thewomens.org.au/health-information/womens-cancers-pre-cancers/cervical-cancer |
| 102 | Ovarian cancer | <https://www.thewomens.org.au/health-information/womens-cancers-pre-cancers/ovarian-cancer> |
| Mater (Hospital/Service/Specialist) | | |
| 103 | Ovarian cancer | <https://www.mater.org.au/health/services/ovarian-cancer> |
| Hunter Valley Oncology (Hospital/Service/Specialist) | | |
| 104 | Ovarian cancer treatment Newcastle | <https://huntervalleyoncology.com.au/services/ovarian-cancer/> |
| Icon Cancer Centre (Hospital/Service/Specialist) | | |
| 105 | Cervical cancer | https://iconcancercentre.com.au/cancer/cervical/ |
| Cherbourg Regional Aboriginal and Islander Community Controlled Health Service (Hospital/Service/Specialist) | | |
| 106 | Ovarian cancer: symptoms, diagnosis and treatment | <https://www.craicchs.org/ovarian-cancer-symptoms-diagnosis-and-treatment/> |
| Globe Medical (Hospital/Service/Specialist) | | |
| 107 | Ovarian cancer | <https://www.globemedical.com.au/adelaide/interact/blog/ovarian-cancer-awareness.html> |
| Gynae-oncologist (Hospital/Service/Specialist) | | |
| 108 | Cervical cancer | <https://www.obermair.info/medical-conditions/gynaecological-cancer/cervical-cancer/> |
| 109 | Cervical cancer | <https://drarora.com.au/gynaecological-cancers/cervical-cancer/> |
| *Abbreviations:* GP: General Practitioner; No.: Number. | | |
